# Supplementary material for: Exploiting the Potential of Magnetic Nanoparticles for Rapid Diagnosis Tests (RDTs): Nanoparticle-Antibody Conjugates and Color Development Strategies
Source: Diagnostics (Basel). 2023 Sep 23;13(19):3033. doi: 10.3390/diagnostics13193033 (PMC10572869; doi:10.3390/diagnostics13193033)
Supplement: Supplementary file 1 [file diagnostics-13-03033-s001.zip › diagnostics-2560771-supplementary.pdf]

diagnostics

MDPI

Supplementary Material

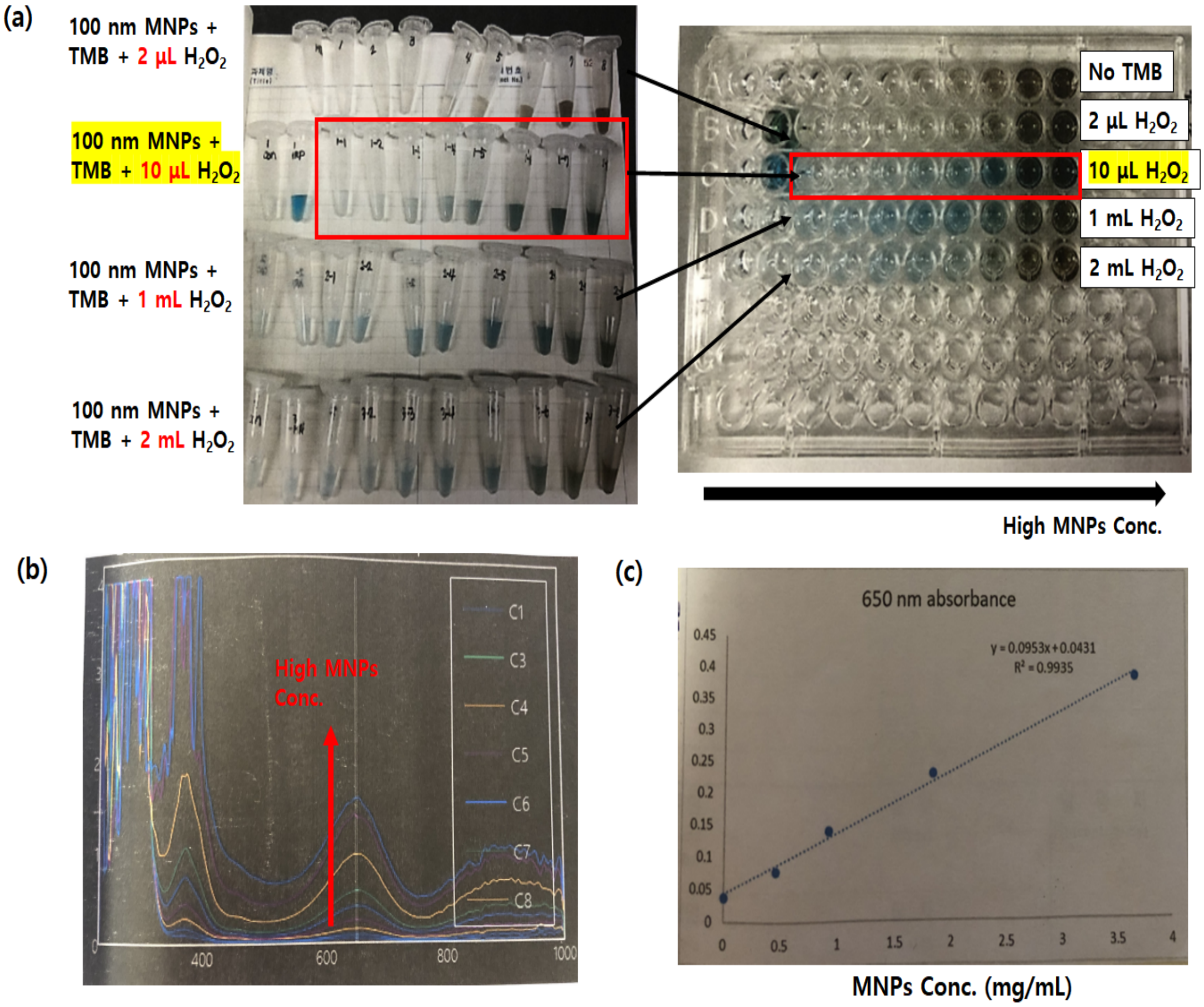

Figure S1. Implementation of color development utilizing the peroxidase-like activity of MNPs (**a**) Visualization of color development of MNPs under varying concentrations of H_2O_2 (b) Increase in 650 nm absorbance with respect to the concentration of MNPs under optimized conditions (**c**) Linear increase in 650 nm absorbance with increasing concentration of MNPs within the range of 0–3.5 mg mL^{-1} under optimized conditions.
